# PRIEST: predicting viral mutations with immune escape capability of SARS-CoV-2 using temporal evolutionary information

**DOI:** 10.1093/bib/bbae218

**Published:** 2024-05-13

**Authors:** Gourab Saha, Shashata Sawmya, Arpita Saha, Md Ajwad Akil, Sadia Tasnim, Md Saifur Rahman, M Sohel Rahman

**Affiliations:** Department of Computer Science and Engineering, Bangladesh University of Engineering and Technology, Dhaka, Bangladesh; Department of Computer Science and Engineering, Bangladesh University of Engineering and Technology, Dhaka, Bangladesh; Department of Computer Science and Engineering, Bangladesh University of Engineering and Technology, Dhaka, Bangladesh; Department of Computer Science and Engineering, Bangladesh University of Engineering and Technology, Dhaka, Bangladesh; Department of Computer Science and Engineering, Bangladesh University of Engineering and Technology, Dhaka, Bangladesh; Department of Computer Science and Engineering, Bangladesh University of Engineering and Technology, Dhaka, Bangladesh; Department of Computer Science and Engineering, Bangladesh University of Engineering and Technology, Dhaka, Bangladesh

**Keywords:** SARS-CoV-2, viral mutations, immune escape, mutation prediction, predictive modeling, generative biological AI

## Abstract

The dynamic evolution of the severe acute respiratory syndrome coronavirus 2 virus is primarily driven by mutations in its genetic sequence, culminating in the emergence of variants with increased capability to evade host immune responses. Accurate prediction of such mutations is fundamental in mitigating pandemic spread and developing effective control measures. This study introduces a robust and interpretable deep-learning approach called PRIEST. This innovative model leverages time-series viral sequences to foresee potential viral mutations. Our comprehensive experimental evaluations underscore PRIEST’s proficiency in accurately predicting immune-evading mutations. Our work represents a substantial step in utilizing deep-learning methodologies for anticipatory viral mutation analysis and pandemic response.

## INTRODUCTION

The severe acute respiratory syndrome coronavirus 2 (SARS-CoV-2) virus had swept the world, causing a global pandemic and the unprecedented COVID-19 crisis. While humanity seems to have survived the worst situation, COVID-19 still impacts worldwide health and society. Viral diseases like COVID-19 are caused by the rapid evolution and virulence of the virus (in this case, SARS-CoV-2). We can follow the evolution and transmission of a virus by identifying and comprehending the genetic changes it undergoes in time. This is crucial, particularly to facilitate vaccine discovery and therapeutics, as we need to develop methods capable of predicting future variants that are powerful enough to escape immunity.

With the rise of more sophisticated high-throughput sequencing technologies and tremendous progress in machine learning techniques in different fields, various effective computational methods have been developed and proposed to analyze viral evolution and predict viral escape. Salama *et al*. [1] proposed a neural network-based model to predict the point mutation by aligning the primary RNA sequence of the Newcastle virus. Yin *et al*. [2] modeled sequential data of the influenza A virus in the form of time series data and used the long short-term memory (LSTM) network with attention to predict mutation at any specific residue site. Takwa *et al*. [3] used a sequence-to-sequence LSTM–recurrent neural network (RNN) model to predict mutations in RNA sequence evolution for successive generations and presented a proof of concept by applying their method on two datasets: Newcastle Disease Virus and influenza virus.

Despite being a relatively newer candidate, the progress on this front with respect to SARS-Cov-2 is also fascinating. Bai *et al*. [4] developed a coarse-grained model and calculated the change of free energy of different single-site or combined-site mutations and predicted potential mutation sites of SARS-CoV-2. Sawmya *et al*. [5] used a mixture of traditional machine learning and deep-learning models for identifying virulence of the genome sequence of SARS-CoV-2 and also used convolutional neural network (CNN)–RNN-based models to predict mutations at particular sites of interest of the genome sequences. Maher *et al*. [6] developed a pipeline using different methods, such as a bidirectional LSTM model, to predict which individual amino acid mutations in SARS-CoV-2 would become more prevalent and contribute to future variants. Zhou *et al*. [7] used phylogenetic tree-based sampling methods of SARS-CoV-2 viral sequences combined with temporal information and a transformer model to predict mutation at specific sites. Similar phylogenetic works have been conducted to trace the spread of the virus [8]. Besides mutation prediction, substantial progress has also been noticed in the literature on viral escape prediction with probabilistic approaches and deep-learning-based methods. Hie *et al*. [9] used Bidirectional LSTM models to identify viral escape mutations of influenza hemagglutinin, HIV-1 envelope glycoprotein (HIV Env) and SARS-CoV-2 Spike viral proteins. Thadani *et al*. [10] introduced an interpretable framework incorporating evolutionary data, structural features and residue dissimilarity properties to predict viral immune escape of SARS-CoV-2. In this work, we developed a novel, interpretable and explainable deep-learning framework using transformer attention mechanism [11] alongside other techniques to effectively predict mutations in the SARS-CoV-2 virus. We validated our predictions by predicting variants ahead of their time. In particular, we corroborated our model’s efficacy by preemptively predicting the emergence of real-world variants ahead of time.

Moreover, our predicted variants were virulent enough to escape immunity, which is a great concern and demands further (wet lab) experimental validation. We argue that our method can be applied to predict mutations at specific sites just from sequence data alone, and it performs competitively with existing methods from a machine learning perspective. Thus, the key contributions of this study are 2-fold:

(1) An interpretable and explainable model with high efficacy in predicting mutations in the SARS-CoV-2 virus that can also be applied in other scenarios, i.e. evolution in other viruses.
(2) A methodology that does not restrict itself to known evolutionary paths and can generalize well enough to never-before-seen cases.

## METHODS

### Dataset preparation

#### Dataset collection and preprocessing

For this study, we used sequences of SARS-CoV-2 available in GISAID [12]. We collected spike protein sequences of the SARS-CoV-2 virus between the final quarter of 2019 and the starting half of 2022. We extracted the primary sequences and Pango lineage: a method developed to label emerging variants in pandemic [13] information from their database. We divided the sequences into three primary classes based on their variants as follows: (i) variants being monitored (VbM), (ii) variants of concern (VoC) and (iii) other variants (i.e. the ones whose Pango Lineage did not fall under any of the known variants that were marked deadly at different points in time during the COVID-19 pandemic). They are labeled by variants 1, 2 and 0, respectively (additional information about this grouping can be found in supplementary section 1).

Our preprocessing steps are inspired by that of Tempel [2], which presents a mutation prediction pipeline for influenza A viruses. However, unlike Tempel, which could leverage 26 years of influenza dataset (containing HA proteins of subtypes H1N1, H3N2 and H5N1), we had SARS-CoV-2 protein sequence data spanning a few years only, i.e. for the period 2019–2022. Therefore, we redesigned the dataset preparation methodology significantly. We resorted to using quarters as our timesteps. Thus, we have four quarters in a year with Qn representing the $n$th quarter (i.e. Q1: January–March, Q2: April–June, Q3: July–September, Q4: October–December). Breaking down the timesteps of each year into quarters led to a total of 10 timesteps. This adjustment had two major impacts on our study. The quarter-level granularity allowed us to construct longer time series, thereby improving the training procedure and increasing the performance of PRIEST to detect mutations. Additionally, shorter timesteps allowed us to tackle the challenges of SARS-CoV-2’s rapid mutations [6, 14–17] (additional details of this timestep-wise division can be found in Supplementary Table 2).

The sequences were of varying lengths but generally comprised more than 1000 amino acid residues. Performing multiple sequence alignment on strings with such length and quantity is very slow and computationally expensive (particularly with the resource-constraint setting we have to work on). Heuristic methods for MSA are faster but are error-prone [18, 19] and would introduce errors in our input data. Thus, we opted for padding the protein sequences. The length of most of the sequences is below 1280. As such, we marked the ones greater than 1280 as outliers and excluded them from further consideration. All the mutation sites of concern are contained within the first 1228 residues. Hence, sequences that were less than 1230 were also removed to ensure all mutation sites were present in each sequence. We padded out the rest of the sequences, which built our library to convert them into sequences of uniform length. Thus, we added a maximum of 50 amino acid residues to our sequence (approximately 4% of the total sequence length). This process of padding did not introduce any additional noise or error, as the mutation sites of concern were not modified.

#### Time series construction

At each timestep, we grouped the protein sequences by the wider classes of different SARS-CoV-2 variants. Since no evidence suggests that variants mutate into older variants, we assume the same in our study. However, the exact nature of SARS-CoV-2 evolution is still unknown. We should also note that the existence of such cases (mutating into older variants) can be easily handled in PRIEST. Our study aims to understand possible changes in the current variants that may lead to next-generation variants. Moreover, the shift in the percentage of each variant collected over time reinforces our assumption about the virus mutation. Therefore we constructed the time series by linking these protein sequences under special conditions as stipulated below.

Let $\ell _{i}$ and $\ell _{i+1}$ be the labels of the protein sequences where $\ell _{i}, \ell _{i+1} \in \{0,1,2\}$. The groups of sequences with label $\ell _{i}$ can be linked to the groups of sequences with label $\ell _{i+1}$ if and only if $\ell _{i} \leq \ell _{i+1}$. This constructs a graph. From now on, we will refer to this as time series graph.

**Figure 1:**
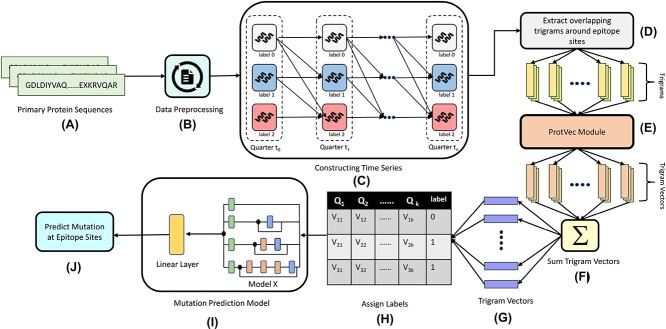
An overview of our mutation prediction pipeline. (**A**) and (**B**) indicate protein sequence collection, clean up and required preprocessing; (**C**) shows time series construction by modeling the time series in terms of quarters from the fourth quarter of 2021 to the fourth quarter of 2022; (**D**)–(**G**) represent steps to create training data for PRIEST; we transformed the training sequences into embeddings that can be used for training. In (**H**) we assigned the labels to our training data; (**I**) indicates training PRIEST and (**J**) represents the mutation prediction at respective epitope sites.

At each timestep, we randomly sample protein sequences from all possible allowed variants. However, whether we can sample from a variant group depends on which group we sampled from in the previous timestep. For example, if we sample a sequence from Variants Being Monitored (VbMs) at timestep $i$, we can only sample from Variants being Monitored (VbMs) and Variants of Concern (VoCs) at timestep $i+1$. We can ensure this by following the links in the time series graph. In addition, we performed this sampling with replacement to cover for the lack of enough data of a specific variant at certain timesteps. For instance, at timestep, $i$, the probability of picking a protein sequence of a certain variant is weighted by the number of samples in the allowed group of sequences at any given timestep. This weighted sampling allows us to focus on the dominant variants at each timestep while keeping the others relevant. Eventually, this allows us to capture the variants’ state better. Figure 1 shows the process of constructing the time series along with the overall mutation prediction pipeline of PRIEST.

#### Input representation

**Figure 2:**
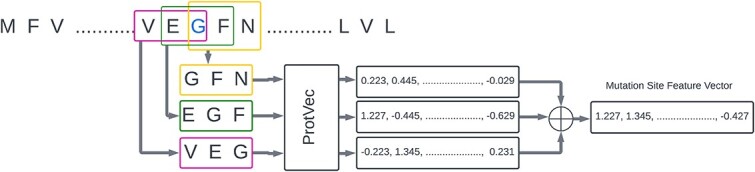
Extraction of feature representation of any given mutation site. We use a sliding window to extract three trigrams around that position. In the sequence above, to get the feature representation of the site marked with blue color, we extract VEG (in red), EGF (in green) and GFN (in yellow). We use ProtVec to extract the feature representation of each of the three trigrams. We aggregate them to obtain the feature representation of the position.

Now that we have our time series data, we need to convert these into trainable data for our deep-learning models. For any given protein sequence time series $\mathcal{S}$ and mutation site $m$, we take the three overlapping trigrams $t_{1}, t_{2}\ and t_{3}$ containing the mutation site $m$ such that $t_{3}, t_{2}$ and $t_{1}$ contain $m$ at the first, second and third positions, respectively (Figure 2). Let us assume we are constructing the time series for site 484, and the protein sequence in Figure 2 is the sequence for timestep $\mathcal{T}$. The trigrams that contain the site are VEF (in red), EGF (in green) and GFN (in yellow). We then take the respective ProtVec [20] representations of the trigrams ($vec_{t_{n}}$). The size ($dim_{vec}$) of each vector of the trigrams is 100. We aggregate them to get the representation of that specific mutation site at timestep $\mathcal{T}$. As such, the time series of vector representations at a particular mutation site $m$ for a given protein sequence time series $\mathcal{S}$ that will be input to PRIEST can be represented by $x_{\mathcal{S}m} \in \mathbb{R}^{k\times dim_{vec}}$, where $k$ = the number of time steps and $dim_{vec} = 100$ and $x_{\mathcal{S}m_{\tau }} = \sum _{n=1}^{3} vec_{t_{n}}$.

#### ProtVec

ProtVec [20] is a sophisticated computational framework inspired by the Word2Vec [21] model, specifically utilizing the Skip-gram [22] architecture to analyze protein sequences. Similar to Word2Vec’s approach to natural language processing, where words are transformed into vector spaces based on their contextual relevance, ProtVec applies this concept to the sequences of amino acids in proteins. By treating amino acids and their respective subsequences (n-grams) as the equivalent of words and phrases, ProtVec is able to capture the contextual relationships within protein sequences. Each amino acid and its n-grams are embedded into a high-dimensional vector space, where the spatial relationships between vectors reflect the biological and functional similarities between the amino acid subsequences. The Skip-gram model is particularly leveraged to predict the presence of certain amino acids given their surrounding sequence context, facilitating the identification of functionally relevant patterns that are not immediately apparent from the primary sequence alone.

#### Label assigning

To train PRIEST, we need to assign a label to each data point. If there is a difference of residue at site $m$ between protein sequences at the final and the penultimate time steps for a protein sequence time series $\mathcal{S}$, we consider this as a mutation and assign a label $1$ to the corresponding time series of vector representations. Otherwise, we assign a label $0$. We construct the dataset with time steps 3, 6 and 9. (The count of positive and negative data points for training, validation and testing set for time steps 3, 6 and 9 are reported in Supplementary Table 3).

### Overview of PRIEST

#### The conv1d block

One of the key components of PRIEST is the Conv1D block. This block takes the vector representation of a time series at a mutation site $x \in \mathbb{R}^{k\times dim_{vec}}$ as input and passes it through a 1D convolution layer [23] of kernel size $k$ and padding $p$ as seen in Equation 1. This is followed by a ReLU [24] activation function and a subsequent dropout [25] layer. Additionally, we applied batch normalization [26] to prevent internal covariance shift. We obtain $x_{conved} \in \mathbb{R}^{k\times dim_{vec}}$ as output. These transformations can be represented by Equation 2. 


(1) \begin{align*} x^{\prime} &= Conv_{(k,p)}(x) \end{align*}

(2) \begin{align*} x^{\prime\prime} &= ReLU(x^{\prime}) \nonumber \\ x^{\prime\prime\prime} &= Dropout(x^{\prime\prime}) \\ x_{conved} &= BatchNorm(x^{\prime\prime\prime}) \nonumber \end{align*}

#### The transformer attention block

The second component is the transformer attention block. This takes inspiration from the self-attention module introduced in [27]. Positional encoding [11] is applied to our input to introduce relative and absolute positional information about the timesteps and can be represented using the Equations 3 and 4. 


(3) \begin{align*} PE_{(\tau, 2i)} = sin({\tau/10\,000^{{2i}/dim_{vec}}}) \nonumber \\ PE_{(\tau, 2i+1)} = cos({\tau/10\,000^{{2i}/dim_{vec}}}) \end{align*}

(4) \begin{align*} x_{out} = x_{in} + PE \end{align*}

The transformed input $x_{PE} \in \mathbb{R}^{k\times dim_{vec}}$ is passed through a transformer encoder [11] with $\phi $ layers and $h$ attention heads. We used an attention mask that prevents the timesteps of the input time series from attending to future timesteps. The rationale behind the mask is that we do not want PRIEST to make decisions about past timesteps using the information of future ones. The output is represented using $x_{TE} \in \mathbb{R}^{k\times dim_{vec}}$. We multiply the output of the transformer encoder by $\alpha $ and the original input by $1-\alpha $ to obtain the output $x_{TA}$. Here, $\alpha $ is a learnable parameter. 


(5) \begin{gather*} x_{TE} = TransformerEncoder_{(\phi,h)}(x_{PE}) \end{gather*}

(6) \begin{gather*} x_{TA} = \alpha x_{TE} + (1-\alpha) x_{in} \end{gather*}

#### PRIEST architecture

**Figure 3:**
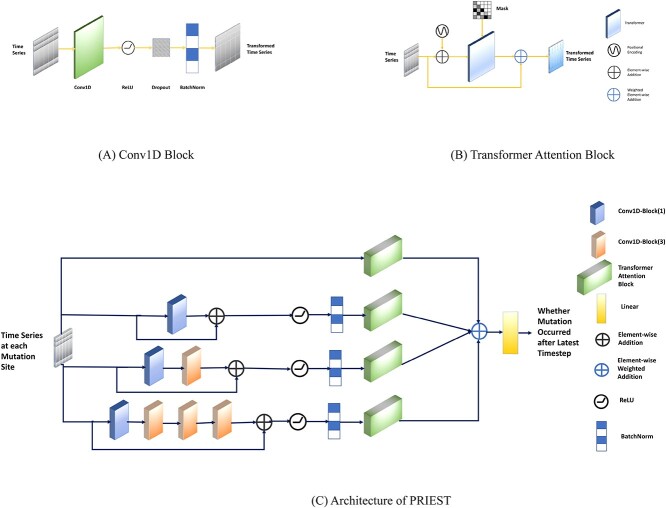
PRIEST architecture with detailed view of all the components. (**A**) The architecture of the 1D convolution block is used for feature transformation. (**B**) The architecture of the transformer attention block. (**C**) A schematic diagram of the overall architecture of PRIEST.

PRIEST incorporates the ideas of the Inception module introduced in [28] and the modifications introduced in [29]. Figure 3 provides detailed illustrations of the various components within the PRIEST architecture. There are four channels. The first channel applies no convolution operations. The second channel applies one convolution operation with a Conv1D Block of kernel size 1. In the third channel, we apply two Conv1D Block operations, one of kernel size 1 and the second of kernel size 3. Finally, the fourth channel applies four consecutive Conv1D Block operations with kernel sizes 1, 3, 3 and 3, respectively. We add the input $x_{in}$ to the output of each of the convolution channels via a residual connection [30]. We apply ReLU and Batch Normalization after the residual connections. We apply the transformer attention mechanism described previously to each of the channels. We represent the outputs by $x_{channel_{i}}$, where $i \in [1,4]$. We multiply them by weights $w_{i}$ and add them to obtain $x_{out}$ (Equation 7). 


(7) \begin{gather*} x_{out} = \sum_{n=1}^{4} x_{channel_{i}} w_{i} \end{gather*}

We extracted the most recent timesteps of $x_{out}$ and passed them through a linear layer to calculate the predicted score, $\hat{y}$. We calculated the cross-entropy loss between ground truth $y$ and $\hat{y}$ and this can be represented by Equation 8. 


(8) \begin{gather*} \begin{split} L = -\frac{1}{NM}\sum_{t=1}^{N}{\sum_{i=1}^{M} \left\{y_{(m_{i})}^{t}log(\widehat{y_{(m_{i})}^{t}}) +(1-y_{(m_{i})}^{t})log(1-\widehat{y_{(m_{i})}^{t}})\right\}} \\ + \lambda \sum_{w \in \mathcal{W}}{w^{2}} \end{split} \end{gather*}

Here, $N$ is the number of training examples and $M$ is the number of mutation sites. Additionally, $m_{i}$ represents $i$-th mutation site. The second term in the loss function is the L2-regularization term. In this term, $\lambda $ is the regularization parameter and it is a hyperparameter, i.e. it is not a learnable parameter. On the other hand, $\mathcal{W}$ represents the set of learnable parameters.

### Mutation quality testing

To validate the effectiveness of PRIEST, we also generated new mutated sequences for Q2 2022 by mutating sequences of Q1 2022. We selected the mutation sites following [31, 32]. Then we used PRIEST to predict mutation at those specific sites for Q1 2022 sequences. In any given mutation site, often more than one residue can replace the existing one. We performed this replacement with equal probability among the possible replacement residues. To understand the strength and broad classification of the newly mutated sequences, we classified the newly mutated sequences using the classification pipeline we developed to classify the three variants from sequence data (additional details related to the classification experiment can be found in supplementary section 4). The sequences that were classified as VoCs are used for further analysis. We represent the set of real-life sequences of Q2 2022 by $Seq_{true}$ and generated sequences by $Seq_{gen}$.

To assess the quality of the generated sequences, we performed four experiments. These experiments are briefed in the following sections.

#### Calculating sequence embedding distance

We calculated the mean of the ProT5 vector representations of the actual Q2 sequences and generated Q2 sequences, represented by ${mean}_{true}$ and ${mean}_{gen}$, respectively. Then we used Euclidean distance and cosine dissimilarity to calculate the distance between the two vectors.

#### Clustering and pairwise distance computation

Often in a set of similar entities, there are subtle differences between individual elements. This is true for SARS-CoV-2 spike proteins. In our study, each larger group of variants consists of one or more variants with multiple lineages. It is often essential that we understand these subtle variances for studies of immunology and antibiotics. Clustering algorithms have proved themselves useful in various tasks in proteomics [33–36], including understanding the underlying differences in protein sequences [37]. To capture these differences in our test sequences, we applied a simple Gaussian Mixture Model (GMM)-based clustering algorithm. Afterward, we computed the pairwise distance between the clusters to capture the details of the difference even further.

For clustering, we first ran principle component analysis (PCA) on the real-life sequences to find the principal components. The two components with the highest degree of variance (details of PCA can be found in supplementary section 7) were picked for downstream analysis. We took these components and divided the sequences into clusters using GMMs. The rationale behind choosing GMM over other clustering algorithms is that it allowed us to determine both the clusters and distribution simultaneously. We determined the number of clusters and covariance types for GMM to be seven and diagonal, respectively (details for GMM hyperparameter selection can be found in supplementary section 8).

We trained a GMM model with the determined hyper-parameters using the first two principal components of the original sequences. Then we fitted our generated sequence’s first two principal components into the clusters. For a quantitative comparison of the clusters, we used the NearestCentroid algorithm [38] to fit the original sequences and the generated sequences with their respective cluster labels. Then we compared the centroids considering both Euclidean distance and cosine similarity.

#### Calculating KL divergence score

So far our assessments have been between the real-world and generated sequences. We now extend our comparison to a more generalized aspect, namely, to the underlying distribution of the two sets. KL-divergence is a very useful metric to evaluate the (dis)similarity between two distributions and we used that to assess the similarity between the distributions of the clusters of the existing and generated sequences. To calculate the KL divergence score, we selected the embeddings of real-life and our generated Q2 2022 sequences. We considered the distribution of original sequence embeddings, $C_{e}$, as our reference distribution and the one for the generated embeddings $C_{g}$ as our approximation for any given cluster. The full algorithm for computing the divergence score is given in Algorithm 1.


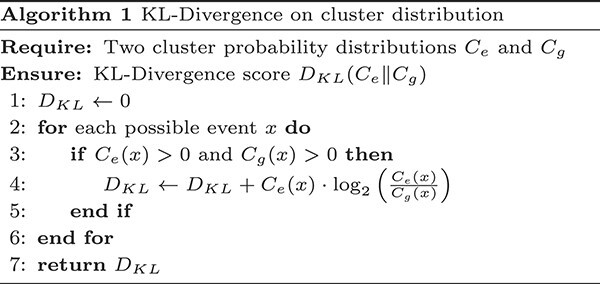


#### Calculating immunescape score

We also experimented with models that can quantify how much a mutated sequence is likely to escape the immune system of the host. We used the EVEScape index introduced in [10], which incorporates fitness predictions from evolutionary models and features related to structural information to determine antibody binding to the mutated proteins, as well as the distance between normal and mutated residues. It uses a probabilistic model that generates a log-likelihood score, giving us the probability of a single amino acid substitution leading to immune or antibody escape. Three separate (likelihood) scores are calculated first as follows [10], and the final score is calculated as the product of these three.

The first score gives the likelihood of maintaining the mutation’s fitness by training a deep generative model. The second score gives the likelihood of the mutation being accessible to the antibody. The third score gives the likelihood of the mutation disrupting binding to the host antibody.

Aside from the original sequences and the mutated sequences generated by PRIEST, we also generated random mutations by modifying the original sequences. For each mutation site, we replaced the existing residue at a mutation site with an alternative with random probability $P$ where each replacement had an equal probability of substitution. Finally, we calculated the respective immunescape scores for both the random mutations and PRIEST-predicted mutations using the aforementioned EVEScape model and compared them.

### Evaluation metrics

In this study, we perform our evaluation from two different angles. First, we evaluate our model PRIEST to compare it with other models from a machine learning point of view. In parallel, we also want to examine the quality of mutations generated by PRIEST. So, we use (i) Model Evaluation Metrics and (ii) Mutation Quality Testing Metrics as described below.

#### Model evaluation metrics

For evaluating PRIEST and other baseline models, we used the Matthews Correlation Coefficient (MCC) and F-score, Receiver Operating Characteristics (ROC) curve and Precision-Recall (PR) curve. For the ROC and PR curves, we consider the area under these curves also known as AUROC and AP, respectively. We consider MCC the primary metric of concern as it provides a better measure of the performance of a model for binary classification over accuracy and F-score, particularly in the presence of data imbalance [39]. In our training, validation and test sets, the number of positive classes outweighs that of negative classes in a 3:1 ratio. Hence, MCC is a good performance metric to assess PRIEST’s performance. We also use F-score as our secondary metric for this particular concern.

#### Mutation quality testing metrics

As detailed in section 2.3, we performed additional tests to verify the quality of mutations generated by PRIEST. For calculating the difference between the generated and real-life sequences in Q2 2022, we used Euclidean distance and cosine dissimilarity. Additionally, we used the KL divergence score to find the difference between the distributions of clusters of similar types of sequences in the generated and real-life sequences. Finally, we used the EVEScape score to assess the chance of our generated sequences to evaluate their potential threats.

### Implementation and training

We used the CNN [40], RNN [41], LSTM [42], gated recurrent unit (GRU) [43] and Tempel (both in temporal attention and dual-attention forms) and Tempo [7] baselines.

The CNN architecture comprised 1D convolution layers followed by 1D max pooling layers. We used three windows of sizes 1, 2 and 3 for convolution and a dropout rate of 0.2. For the remaining baselines, a hidden size of 128 and a dropout rate of 0.5 were used. We used Tempo with two transformer encoder layers and five attention heads.

In PRIEST, we used 1D convolution layers of kernel size 1 and padding size 0, and kernel size 3 and padding size 1. Additionally, transformer encoders in PRIEST had two transformer encoder layers and five attention heads. We applied a dropout of 0.5 to all layers and 0.1 in positional encoding.

To maintain consistency in performance measurement, we trained all the models for 50 epochs with a batch size of 256. We used Adam [44] as our optimizer with learning rate ${1}\times 10^{-3}$. To prevent overfitting and ensure an additional boost to our performance, we used a cyclic learning rate scheduler [19].

### Environment and hardware

We used Pytorch and Scikit-learn to implement all the architectures and evaluate metrics. All training and experiments were done on a machine with Intel Core™ i7-9700F CPU @ 3.00GHz processor with eight cores and 16GB of RAM, and GeForce RTX 2070 SUPER GPU with 8GB VRAM and Ubuntu 22.04.2.

## RESULTS AND DISCUSSION

### Efficacy of mutation prediction

The performances of all the models are reported in Table 1. As we can see PRIEST has a higher MCC than other baseline models for $k \in \{3, 6\}$. For $k=9$, it still has comparable performance. Moreover, with respect to F-Score, PRIEST outperforms or achieves results similar to those of other models across all the different time series sequence lengths.

**Table 1:** A comparison of evaluation metrics (MCC and F-Score) obtained by PRIEST and other benchmark models on independent test set

	MCC			F-Score		
Model	$\boldsymbol{k=3}$	$\boldsymbol{k=6}$	$\boldsymbol{k=9}$	$\boldsymbol{k=3}$	$\boldsymbol{k=6}$	$\boldsymbol{k=9}$
RNN	0.651	0.709	0.695	0.918	0.918	0.919
LSTM	0.695	0.729	0.730	**0.925**	0.923	0.926
GRU	0.695	0.728	0.734	**0.925**	0.922	**0.927**
Tempel (DA-RNN)	0.512	0.611	0.657	0.898	0.898	0.910
Tempel (Attention)	0.695	0.734	**0.735**	**0.925**	0.924	**0.927**
CNN	0.648	0.709	0.713	0.919	0.919	0.922
TEMPO	0.683	0.740	0.732	0.921	0.925	0.926
PRIEST	**0.699**	**0.747**	0.730	**0.925**	**0.927**	0.926

Elements in bold represent the highest value for any given metric for a specific value of $k$.

Tempel uses LSTM as hidden units. While LSTMs outperform GRUs and RNNs in capturing long-term dependency, they are still outperformed by transformers in capturing sequential/temporal information. As these units are sequential, a loss of information is expected with RNN and similar recurrence-based models. Transformers use self-attention. Instead of looking at the timesteps in a recurrent manner, it looks at the whole time series. As such, it is generally more potent in capturing long-term dependencies.

While Tempo only leveraged this power of transformers and the attention mechanism that comes with it, PRIEST leverages the power of 1D convolution layers to extract additional features. It uses trainable weight parameters to focus on the extracted features or original time series as required.

The performance of PRIEST deteriorates when $k = 9$. Tempel outperforms PRIEST in that specific case albeit marginally. Here, loss of information due to the inability to capture long-term dependency is actually helpful for Tempel. The longer series might add additional information that is not quite as useful for predictions. On the other hand, Temple might have lost the information about the earlier timesteps and only used the useful ones. Ultimately, the model’s complexity, while benefiting PRIEST in most cases, actually hurts in this particular one.

**Figure 4:**
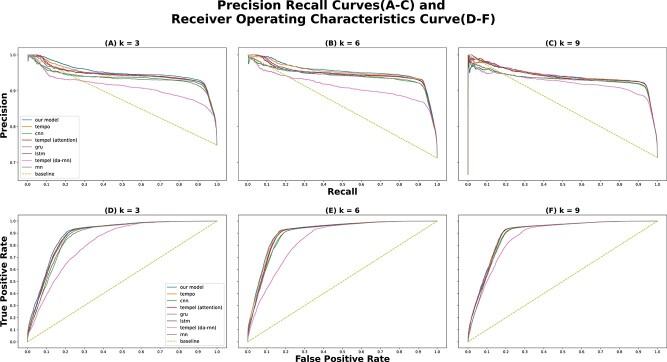
The PR (A-C) and ROC (D-F) curves for PRIEST and other baseline models for timesteps $k \in \{3, 6, 9\}$. PRIEST comfortably outperforms the other models by having both greater AP and AUROC, respectively.

In terms of AUROC and AP, PRIEST outperforms all other baseline models. This is evident in Figure 4 where we plotted the PR and ROC curves; PRIEST has a higher area under both the curves in all cases $k \in \{3, 6, 9\}$ (detailed in supplementary section 5). Since PRIEST at $k=6$ outperforms all other models in all scenarios, we will use this for further analysis for the remainder of this study.

#### PRIEST does not require strict grouping

PRIEST does not require any strict grouping of sequences and works with any form of grouping. To this end, we believe it would be best to show the results of similar experiments with sequences from 2023, as the two major groups of variants over the year were VbMs and VoCs.

We can see from Table 2 that PRIEST outperforms other baselines even in this new scenario in regard to our primary metric, MCC. Other metrics are provided in the supplementary section 6.

**Table 2:** Comparison of PRIEST with baselines for 2023 sequences

Model	MCC
RNN	0.281
LSTM	0.340
GRU	0.344
Tempel (DA-RNN )	0.224
Tempel (Attention )	0.328
CNN	0.295
TEMPO	0.323
PRIEST	**0.348**

Elements in bold represent the highest value.

### PRIEST’s efficacy in creating mutated sequences

To test PRIEST’s ability to detect mutation, we tested it on an independent test dataset to predict the mutations that would arise in Q2 2022. We tested the quality of the mutations that were predicted by PRIEST, ran the tests as described in section 2.3. In this section, we are going to describe those results.

#### Embedding similarity

The Euclidean distance between $mean_{true}$ and $mean_{gen}$ was 0.368, while the cosine dissimilarity was 0.024. The results of this study indicate a high level of similarity between the Q2 2022 sequences and the sequences generated in this study, as demonstrated by two different measures.

A lower value of Euclidean distance indicates higher quality as it indicates the closeness of the two sets. The Euclidean distance between the mean values of the two sets of sequences was found to be 0.368, indicating that the two sets of sequences are relatively close to each other in the latent vector space.

Additionally, cosine dissimilarity can vary between 0 (completely similar) and 1 (completely dissimilar). The cosine dissimilarity between the two sets of sequences was measured to be 0.024, indicating a high degree of alignment between the two sets of sequences.

#### Clustering and pairwise distance computation

**Figure 5:**
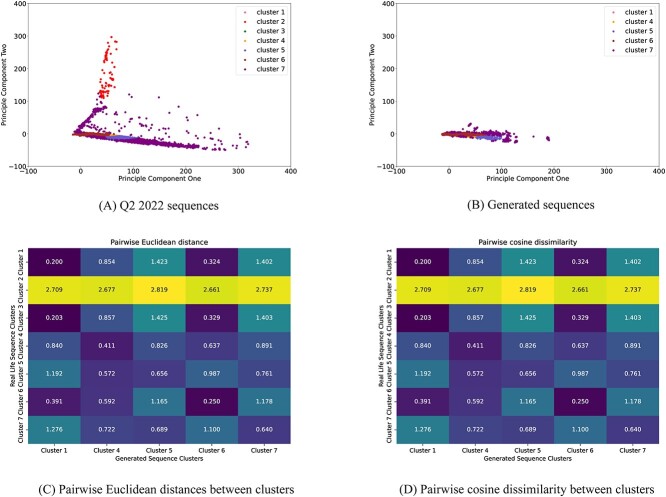
(**A-B**) Cluster comparison of actual and generated sequences. Exact coloring is maintained for sequences belonging to the same clusters in both cases. (**C**) Pairwise Euclidean distance between the cluster centroids (**D**) Pairwise cosine dissimilarity between the cluster centroids.

Our experiments reveal that the generated sequence clusters are highly comparable with the original ones, as seen in Figures 5(A) and 5(B). Most of the clusters present in the set of existing sequences were also observed in the generated set, except for Cluster 4 and Cluster 6.

To further investigate the similarity between the two sets of sequence clusters, Figures 5(C) and 5(D) show the pairwise distances between centroids of each cluster of the actual and generated sequences. The centroids of all the generated sequence clusters were closest to their corresponding existing sequence cluster centroids. Likewise, Figure 5(D) also shows that the centroids of generated sequence clusters were similar to their corresponding actual sequence cluster centroids.

Overall, these findings suggest that the generated and original sequence clusters are indeed close to each other with no anomalies.

#### KL divergence score

To assess the similarity between the distributions of the clusters of the existing and generated sequences, we calculated the KL divergence score. The results presented in Table 3 show that the scores for clusters 1, 4, 5 and 6 are very low, ranging from 0.00001 to 0.066. This suggests that the distributions of the clusters of existing sequences and those of the generated ones are quite similar.

**Table 3:** KL divergence score of clusters

Cluster name	KL divergence score
Cluster 1	${1.517}\times 10^{-5}$
Cluster 4	0.028
Cluster 5	0.066
Cluster 6	0.026
Cluster 7	0.227

However, for cluster 7, the KL divergence score is 0.227, which is higher than the other clusters. This finding is consistent with the observation made in the analysis of the cluster centroids’ distance, as mentioned earlier. These results provide additional evidence that the generated and original sequence clusters are comparable with each other, with only a few exceptions.

Overall, the KL divergence scores indicate that the generated sequence embeddings’ underlying distributions are very close to those of the original sequence embeddings.

#### Immune escape

Next, we compare the average immunescape score for both the random mutations and PRIEST-generated mutations using the EVEScape. The scores for each probability value of random mutations are shown in Table 4. The average immune escape score for mutations generated by PRIEST is $-51.049$. The score is higher than any random mutations, indicating that the mutations generated using PRIEST may escape the host. Additionally, it further shows that the mutations generated by PRIEST are closer to natural mutations.

**Table 4:** Immunescape scores against various random mutation probabilities

Probability of random mutations	Immunescape score of random mutations
0.5	−133.861
0.6	−160.697
0.7	−187.502
0.8	−214.231
0.9	−240.987

## HIGH-PROBABILITY MUTATION SITES

In analyzing the mutations of the virus, we focus primarily on two regions of the spike protein. The spike protein of the SARS-CoV-2 virus plays a pivotal role in the virus’s ability to infect host cells, facilitated primarily through two critical domains: the receptor-binding domain (RBD) and the N-terminal domain (NTD). The RBD is crucial for the virus’s attachment to host cell receptors, specifically the angiotensin-converting enzyme 2, mediating the entry of the virus into the host cell [45, 46]. Meanwhile, the NTD, although less understood, is implicated in processes that might influence virus attachment and entry into host cells [47]. These domains are significant targets for neutralizing antibodies, thus central to the virus’s antigenicity and the host’s immunodominant response [32]. The immunodominance of these domains is attributed to their high mutation rates, leading to varied antigenic properties that can influence vaccine effectiveness and the development of therapeutic antibodies. Understanding the roles of RBD and NTD in immunodominance and antigenicity is crucial for designing effective interventions and predicting the virus’s evolutionary trajectory, especially in response to host immune pressures.

While analyzing viability of PRIEST in detecting meaningful mutations, it is important to analyze the sites which are most critical to PRIEST. We can do this 2-fold. The first of these is to determine which are the most important sites as predicted by PRIEST. To this end, we need to test whether the top sites according to PRIEST fall within the critical regions as discussed above.

Table 5 shows the top 20 sites with the highest mutation probability as predicted by PRIEST. We can see that PRIEST accurately predicts the high-probability mutation sites. Some of the currently known mutations are also present in the table. While mutations that cause immunescape occur in a specific site, the neutralizing antibody (nAb) that causes this immunescape in the first place also interacts with other sites in the vicinity. Thus, those adjacent sites are also prone to mutations [48]. Our model accurately predicts these as potential mutation sites as well. For example, from Table 5, we can see that K417N is a well-known mutation in site 417. However, our model predicts sites 413–416 as potential high-risk mutation sites. Some of these well-known interactions in these sites are D414, D415 and F416 [49]. Similar trends can be seen in sites 444 and 445, with well-known interactions being K444 and V445, and the prominent mutation is seen in sites 446 and G446S. The mutations fall within the RBD region of S-glycoprotein. This is the most well-known mutation region in Omicron Variants, i.e. the VoCs. The alignment of existing evidence with the predictions made by PRIEST gives validity to its predictions. Thus, a site that has been predicted by PRIEST as a high-probability mutation site but has no known mutation associated with it is worth keeping an eye on, as these sites are quite likely to undergo mutations as new lineages/variants emerge.

**Table 5:** Top 20 mutation sites with the respective probability of mutation as calculated by PRIEST

Mutation site	Probability of mutation	Known mutations in Q2 2022
413	0.950	–
808	0.889	–
414	0.778	–
452	0.696	L452(Multiple)
445	0.686	–
459	0.675	–
415	0.670	–
458	0.666	–
416	0.641	–
439	0.634	Q439K, N439(Multiple)
464	0.616	–
444	0.615	–
460	0.597	N460K
446	0.590	G446S
462	0.581	–
461	0.570	–
463	0.553	–
453	0.552	–
417	0.550	K417N
405	0.494	D405N

## INTERPRETATION

Additionally, we wanted to validate PRIEST’s ability to focus on the sites from the most crucial mutation regions while making predictions. As discussed in Section 4, these two regions are RBD and NTD. We indeed can see that PRIEST pays attention to the most relevant mutation sites while making inferences (the process of extracting and aggregating these attention scores is detailed in supplementary section 12). In Q4 2021, the majority of the sites were located around the RBD region: the major mutations found in S-glycoproteins of VoCs [50, 51], while the rest were in the NTD region, the other significant mutation region of S-glycoprotein. A similar trend can be observed in Q2 2021 and Q3 2021. However, the mutation sites were more evenly distributed between the RBD and NTD regions. In timesteps older than Q2 2021, the model primarily focused on sites belonging to the NTD region. The ones which were not part of the NTD region belonged to NSP1 and ORF3. These are two of the major regions outside the S-glycoprotein mutations.

We can conclude from this discussion that during the prediction of mutation, PRIEST pays attention to the mutation sites belonging to the most vital regions in each timestep. This was achieved without ever passing this information explicitly.

## LIMITATIONS AND FUTURE PROSPECTS

PRIEST is a general framework for viral mutation prediction. However, it is not without some limitations. We did not evaluate our predictions in the light of Polygenic Risk Scoring to associate the disease at the individual level. Secondly, PRIEST requires sufficient evolutionary information regarding the virus sequence to generate reasonable predictions. The simple training regime allows PRIEST to make predictions with reasonable dataset size, extending even up to pre-pandemic data. In this work, we focused on working within and post-pandemic data ablating with the limited data available to us. Another limitation of our work is its lack of rigorous biological interpretation. While we have made some effort to interpret our model, further effort may be given to get more biological insight from the model, which we plan to do as an immediate future work.

## CONCLUSION

Mutation prediction in genome sequences is a challenging task at the core of biological research. Moreover, predicting the viral evolution of a dynamic virus such as SARS-CoV-2 requires sophisticated methods that can tread in the shifting constraint of immunity [10]. In this study, we presented a novel state-of-the-art method to predict possible mutations in SARS-CoV-2 using historical mutation information. Our method outperforms the existing methods currently used in this field of study. This allows us to deal with the prediction of viral evolution with greater accuracy. Additionally, we present some unique mathematical studies that allow us to test the quality of the new generation of viruses. Not only can these methods be used for future directions of this study, but will also allow us to test relationships among variants of any virus.

Preparation for an upcoming pandemic is impossible if we are not equipped with an early detection mechanism and tools to predict and simulate the future variants of a quickly evolving ‘germ’ of the pandemic. Furthermore, a robust method should likely detect key escape mutations on the shifting grounds of viral immune escape. PRIEST is a flexible method that incorporates evolutionary information to generate such predictions. This allows us to keep up with this continuously evolving virus and allows doctors and researchers to create vaccines as soon as a new variant arrives. We would no longer need a new variant to be available in nature to create a vaccine for it. This will allow us to save countless lives, something we could not do when COVID-19 broke out. Additionally, the simplifying nature of the assumptions we made allows us to use the method for other viruses and pandemics in general.

**Key Points**

- PRIEST is an interpretable and explainable model with high efficacy in predicting mutations in the SARS-CoV-2 virus that can also be applied in other scenarios, i.e. evolution in other viruses.
- It is a methodology that does not restrict itself to known evolutionary paths and can generalize well enough to never-before-seen cases.
- The sequences generated by PRIEST demonstrate a high likelihood of evading the immune system, which is important for future vaccine design.
- PRIEST can be crucial for early detection and simulation of future variants of rapidly evolving viruses, enhancing pandemic preparedness and response.

## Supplementary Material

PRIEST_Supplementary_Materials_bbae218

## Data Availability

The raw data that were used in this study were obtained from GISAID[12]. The preprocessed data which were used to train and evaluate PRIEST and other baseline models are available at https://drive.google.com/drive/folders/15LPGCb3x_T8uA9eNgK_ClxYdZS_MUeiS?usp=sharing.

## CODE AVAILABILITY

PRIEST is available as an open-source implementation in https://github.com/ComeBackCity/PRIEST. Additionally, the code for EVEScape used in immunescape analysis is available at https://github.com/OATML-Markslab/EVEscape.

## Author Biographies

**Gourab Saha** is a Lecturer at the Department of Computer Science and Engineering at United International University. His research interests include structural and molecular computational biology, biomedical computer vision, and the study of machine learning theories.

**Shashata Sawmya** is a Graduate Research Assistant in the Department of Electrical Engineering and Computer Science at the Massachusetts Institute of Technology. His research interests include computational neurobiology, computational molecular biology, and efficient machine learning.

**Arpita Saha** is a Research Assistant at Brandeis University. Her research interests include efficient privacy-aware data systems, bioinformatics, and medical informatics.

**Md Ajwad Akil** is a graduate student pursuing PhD at the Department of Computer Science at Purdue University. His research interests include the application of machine learning and natural language processing in cybersecurity, software security, and computational biology.

**Sadia Tasnim** is a Software Engineer at Auptimate Technologies Ltd. and an ex-graduate of the Department of Computer Science and Engineering, Bangladesh University of Engineering and Technology (BUET).

**M. Saifur Rahman** is a Professor at the Department of Computer Science and Engineering, Bangladesh University of Engineering and Technology (BUET). His research interests include bioinformatics, health informatics, and algorithms.

**M. Sohel Rahman** is a Professor at the Department of Computer Science and Engineering, Bangladesh University of Engineering and Technology (BUET). His research interests include algorithms, bioinformatics, and metaheuristics.
