# Supplementary material for: PRIEST: predicting viral mutations with immune escape capability of SARS-CoV-2 using temporal evolutionary information
Source: Brief Bioinform. 2024 May 13;25(3):bbae218. doi: 10.1093/bib/bbae218 (PMC11091746; doi:10.1093/bib/bbae218)
Supplement: PRIEST_Supplementary_Materials_bbae218 [file priest_supplementary_materials_bbae218.pdf]

Supplementary information for PRIEST - Predicting viral mutations with immune escape capability of SARS-CoV-2 using temporal evolutionary information

Gourab Saha[1†], Shashata Sawmya[1†], Arpita Saha[1], Md. Ajwad Akil[1], Sadia Tasnim[1], Md. Saifur Rahman[1], M. Sohel Rahman[1*]

[1*]Department of Computer Science and Engineering, Bangladesh University of Enginnering and Technology, Dhaka, Bangladesh.

*Corresponding author(s). E-mail(s): msrahman@cse.buet.ac.bd;
†These authors contributed equally to this work.

1 Grouping variants into larger variant groups that PRIEST uses as class labels for sequences

We collected the protein sequences from GISAID[1]. Among many of the available information, we were particularly interested in the primary sequence, Pango lineage information and collection date. We used the Centers for Disease Control and Prevention(CDC) Database[2] (version of June 2022) to assign each variant its larger class label. The details of this assignment are described in Table 1.

2 Statistics of the protein samples collected

Table 2 shows the number of protein samples collected. They are grouped by quarter of each year.

3 Details of train, test, and validation tests used in training and evaluation of PRIEST and other baselines

Table 3 shows us the number of positive and negative mutations in the datasets we used in training for all values of $k \in \{3, 6, 9\}$.

Table 1: Details of class assignment to each variant alongside their specific Pango lineages used in this study

Variant	Class	Example Lineages
Alpha	Variants being monitored	B.1.1.7, Q
Beta	Variants being monitored	B.1.351.*[a]
Gamma	Variants being monitored	P.1.*[b]
Delta	Variants being monitored	B.1.617.2 & AY
Epsilon	Variants being monitored	B.1.427, B.1.429
Eta	Variants being monitored	B.1.525
Iota	Variants being monitored	B.1.526
Kappa	Variants being monitored	B.1.617.1
-	Variants being monitored	1.617.3[c]
Zeta	Variants being monitored	P.2
Omicron	Variants of concern	B.1.1.529, BA.1, BA.1.1, BA.2, BA.3, BA.4 and BA.5.*[d]

[2] Other variants are the ones that didn't have Pango Lineage information.

[a] 1
[b] 1
[c] The name of this variant was unknown when this data was collected
[d] An asterisk after this lineages means these lineages and their descendants.

Table 2: Number of protein sequences collected per class grouped by years and quarters

Timestep	Year	Quarter	Count by Classes		
			Class 0	Class 1	Class 2
1	2019	4	4	0	0
2	2020	1	7485	32	0
3	2020	2	17654	43	0
4	2020	3	21580	923	0
5	2020	4	38942	8491	0
6	2021	1	67745	57452	5
7	2021	2	23758	109051	1
8	2021	3	6648	254269	1
9	2021	4	4330	200097	53209
10	2022	1	625	2660	38255

Table 3: Details of train, validation, and testing set for time steps $k \in \{3, 6, 9\}$

	k=3		k=6		k=9	
Data Split	0	1	0	1	0	1
Train	128631	318369	127083	319917	125311	321689
Validation	7292	22508	8186	21614	9579	20221
Test	7483	22317	8556	21244	8518	21282

4 Variant Classification

We required a classification model that could help us classify any given sequence into one of VBM, VOC, or other variants. This model was essential for us when we generated the sequences to test the quality of the sequences generated by PRIEST. Our simple but effective MLP-based classification model outperforms other baseline classifiers. The details of the classifier are given below.

4.1 Classifier Architecture

MLP-based classifiers have been used in various computational biology tasks[3–6]. However, as MLP cannot directly process sequences because they are prone to overfitting to the specific patterns present in the training sequences, we needed some vector representation of these sequences. We used a pre-trained ProT5[7] model for this transformation. Each representation is a vector V = (V_0, V_1, ..., V_{1023}) which has a dimension of 1024. We labeled each vector by its sequence labels and passed these through various classifier models. Figure 1 shows an overview of this pipeline.

The variant classifier consists of 6 hidden layers. The first five hidden layers had 512, 256, 64, 32, and 8 activation units, respectively. Each of these layers had a layer of ReLU[8] as non-linearity after it. The final layer had three activation units, each for one of the classes. A Softmax[9] layer follows this.

4.2 Implementation and training

We used Q4 2019 - Q1 2022 sequences to train the classifier. We divided the data into three splits of 80-10-10 with constant seed for consistency of testing across the models. Apart from conversion to embeddings, all other preprocessing steps were done using Scikit-Learn's Preprocessing module. For our baselines, we used gradient-boosted trees found in LightGBM and XGBoost. We used their respective Python libraries. LightGBM and XGBoost are well known for potency in classification across various tasks [10–12] and [13–16] respectively. The various hyperparameters and other training details of other baselines and our variant classifier are shown in Table 4.

4.3 Results of Variant Classifiers

Our primary metrics of concern for classification are Accuracy, F1-Score, and ROC-AUC Score. Table 5 shows that our MLP-based classifier model matches or outperforms other baseline models regarding all scores, including our metrics of concern. This is due to the availability of a large quantity of data. Values of precision and recall metrics are also mentioned.

5 Performance comparison of PRIEST and other baseline models on additional metrics

The performance for other metrics such as accuracy, precision, recall, AUROC, and AP of all the models used in our experiments is reported in Table 6. Regarding accuracy, PRIEST has the best score when $k \in \{3, 6\}$ while comparable to others at $k = 9$. PRIEST has a higher precision value than other baseline models for $k \in \{3, 6\}$. Additionally, for $k = 9$, it still has comparable performance, coming in second. For the AUROC score, PRIEST outperforms for all values of k

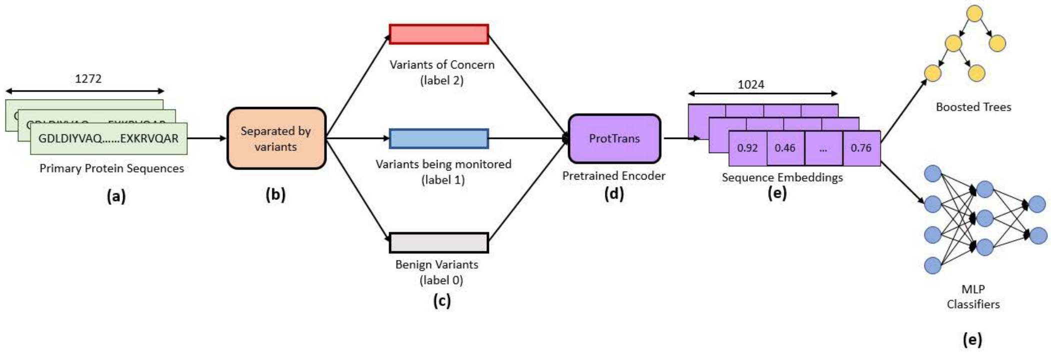

Fig. 1: An overview of the classification pipeline used in this study. (a) shows the collection of the sample spike protein sequences. (b) shows grouping the sequences into larger variant groups. In (c), we label the sequences. (d) shows feeding the sequences into a pre-trained ProT5 model. In step (e), we get the vector representations of the sequences. (f) shows the process of training the classifiers using the sequences and their labels.

Table 4: Hyperparameters used in baseline and variant classifier training for variant classification task

Hyperparameter / Model	LGBM	XGBoost	Variant Classifier
Boosting Rounds	1000	-	-
Objective Function	Softmax	Softmax	Softmax
Loss Function	Multiclass log loss	Multiclass log loss	Categorical cross entropy[a]
Number of Estimators -	100	-	
Max Depth	6	7	-
Learning Rate	-	0.1	0.001
Batch Size	-	-	32
Epochs Trained	-	-	100
Optimizer	-	-	Adam[b]

[3]Hyphens indicate that these hyperparameters are not applicable for that particular model

[4]All models were trained on GPU

[5]The hyperparameters were determined empirically.

[a]Loss uses uses label smoothing with value 0.1

[b]Optimizer uses amsgrad[17] and L2 regularization with weight decay value 0.001

Table 5: Performance of our MLP-based variant classifier and baselines on test set

Model	Accuracy	Precision	Recall	F1-Score	ROC-AUC Score
LGBM	0.95	0.95	0.94	0.95	0.99
XGBoost	**0.97**	0.96	0.96	0.96	**1.00**
ANN	**0.97**	**0.97**	**0.97**	**0.97**	**1.00**

compared to other baseline models. As for the AP score, we see a similar case to the precision score, with a higher AP value for $k \in \{3, 6\}$. For Recall value, PRIEST performs competitively with other baseline models for all k.

6 Performance comparison(additional metrics) of PRIEST and other baseline models on 2023 data

While MCC is our primary metric of concern, we did calculate the other metrics as well for our experiments of 2023. They are mentioned in Table-7.

7 Details of PCA parameter selection

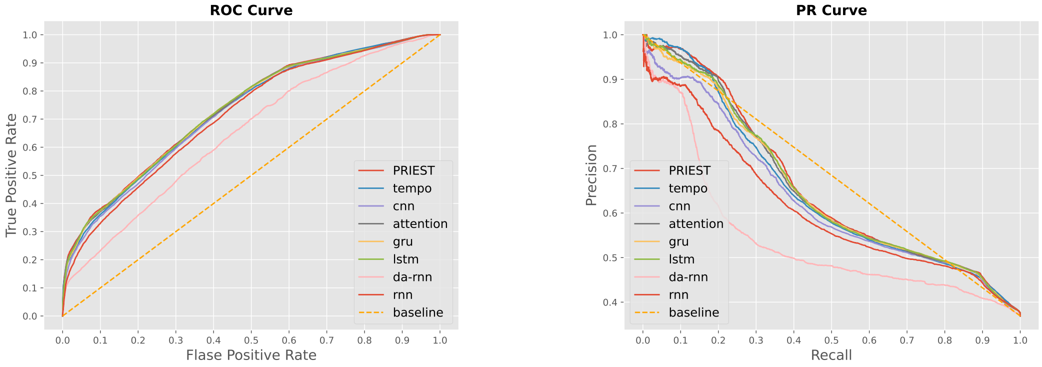

(a) ROC Curve

(b) PR Curve

Fig. 2: ROC and PR curves for PRIEST and other baselines on 2023 data

Figure 3 shows us the variance vs. components plots for both real life Q2 2022 sequences and sequences generated by PRIEST. We can see that the first two PCA components have the highest variances and thus lead to the most impact on the clustering down the line. So we picked these components for our further experiments.

Table (6) A comparison of evaluation metrics (Accuracy, Precision, Recall, AUROC, and AP) obtained by PRIEST and other benchmark models on independent test set

	Accuracy			Precsion			Recall		
Model	3	6	9	3	6	9	3	6	9
RNN	0.875	0.883	0.880	0.894	0.909	0.889	0.944	0.928	**0.950**
LSTM	**0.887**	0.890	0.892	0.918	0.920	0.908	0.932	0.926	0.945
GRU	0.884	0.889	0.894	0.916	0.919	0.910	0.931	0.926	0.945
Tempel(DA-RNN)	0.832	0.848	0.866	0.824	0.861	0.874	**0.987**	**0.939**	0.948
Tempel(Attention)	**0.887**	0.891	**0.894**	0.919	0.923	**0.911**	0.931	0.925	0.944
CNN	0.875	0.883	0.886	0.888	0.908	0.899	0.952	0.930	0.947
TEMPO	0.881	0.894	0.893	0.920	0.926	**0.911**	0.921	0.925	0.942
PRIEST	**0.887**	**0.896**	0.892	**0.924**	**0.929**	0.908	0.925	0.925	0.945

	AUROC			AP		
Model	3	6	9	3	6	9
RNN	0.871	0.898	0.886	0.934	0.941	0.931
LSTM	0.887	0.904	0.894	0.944	0.946	0.939
GRU	0.885	0.901	0.896	0.944	0.944	**0.941**
Tempel(DA-RNN)	0.818	0.846	0.868	0.912	0.912	0.922
Tempel(Attention)	0.884	0.904	0.895	0.944	0.946	0.940
CNN	0.876	0.896	0.888	0.936	0.939	0.931
TEMPO	0.888	0.910	0.897	0.946	0.950	0.940
PRIEST	**0.895**	**0.914**	**0.898**	**0.950**	**0.953**	0.940

Table 7: Additional metrics of PRIEST and other baselines on 2023 data

Model	Accuracy	Precision	Recall	F1-Score	AUROC	AP
RNN	0.686	0.628	0.367	0.463	0.719	0.716
LSTM	0.710	0.713	0.357	0.476	0.777	0.772
GRU	0.711	0.733	0.342	0.466	0.770	0.766
Tempel (DA-RNN)	0.667	**0.796**	0.130	0.223	0.719	0.711
Tempel (Attention)	0.705	0.689	0.368	0.480	0.776	0.773
CNN	0.690	0.620	**0.409**	**0.493**	0.752	0.751
TEMPO	0.704	0.710	0.333	0.454	0.774	0.772
PRIEST	**0.713**	0.720	0.364	0.483	**0.779**	**0.778**

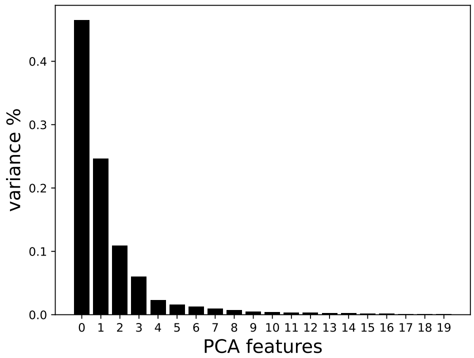

(a) Graph for Existing Sequences of Q2 2022

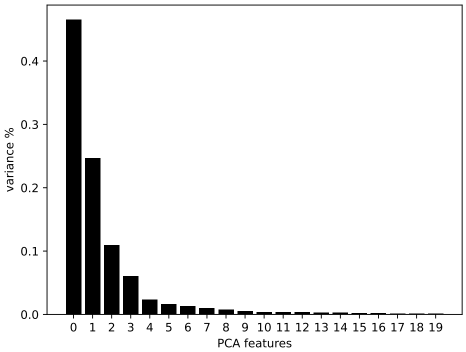

(b) Graph for Generated Sequences of Q2 2022

Fig. 3: Plots for PCA Components Selection

8 Details of GMM parameter selection

GMM uses the Bayesian Information Criterion (BIC) score to identify one hyperparameter, the number of clusters with a lower BIC score being better. The second hyperparameter covariance type controls the degrees of freedom and defines the shape of each cluster. There are four covariance types: spherical, tied, diagonal, and full. To obtain the desired number of clusters and the covariance type, we first used grid search, considering up to nine components and all covariance types. We then generated the BIC score for each covariance type with respect to all the clusters. We observed from the plots of the BIC score vs. the number of clusters that the score for the diagonal type of covariance is the lowest, so this is our desired covariance type. To determine the number of clusters, we calculated the gradients of the BIC score. We observed that the gradient for the diagonal type of covariance did not improve after seven components. Thus, we selected our desired number of components to be seven for performing the clustering.

From graph 4a of BIC score vs. numbers of components of different covariance types, we observe that the plot of BIC score of diagonal type covariance is lower than all other covariance types for components 2, 5, 6, 7, 8, and 9. So we selected this covariance type among all others.

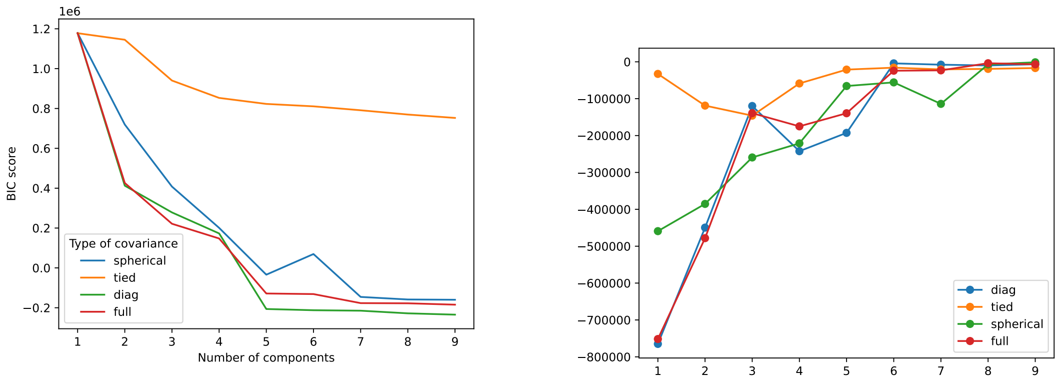

(a) Plots of BIC Score vs. Number of Components for different Coavariance Types

(b) Plots of Gradients of BIC score vs. Number of Components for different Covariance Types

Fig. 4: Plots for GMM Parameter Selection

From graph 4b of Gradients of BIC score vs. the number of components, we observe that the gradient of spherical and full type of covariance changed after seven components. In comparison, the gradient of the diagonal and full type of covariance remained static after seven components. So, we finally fixed the number of components for the diagonal type of covariance to 7. Thus we finalized the covariance type to be diagonal and used 7 clusters or components for our GMM for testing the quality of generated mutated sequences by PRIEST.

9 3D plots of the clustering diagrams

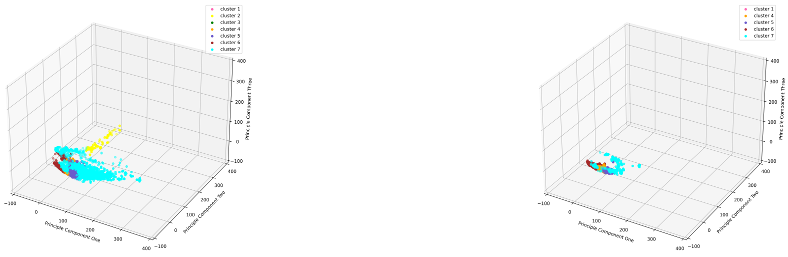

(a) Q2 2022 sequences

(b) Generated sequences

Fig. 5: Cluster comparison of actual and generated sequences. Exact coloring is maintained for sequences belonging to the same clusters in both cases.

10 Details of Euclidean Distance and Cosine Dissimilarity Scores

Table 8a and 8b show the Euclidean distance and Cosine dissimilarity between observed between the sequences in seq_{gen} and seq_{true}.

11 Details of immunescape calculation using EVEscape

We also experimented with models that can quantify how much a mutated sequence is likely to escape the immune system of the host. We used the EVEScape index introduced in [18], which incorporates fitness predictions from evolutionary models, and features related to structural information to determine antibody binding to the mutated proteins, as well as the distance between normal and mutated residues. It uses a probabilistic model that generates a log-likelihood score, giving us the probability of a single amino acid substitution leading to immune or antibody escape. The product of three separate probabilities calculates this score. The first score gives the likelihood of maintaining the mutation's fitness by training a deep generative model. The second score gives the likelihood of the mutation being accessible to the antibody. The third score gives the likelihood of the mutation disrupting binding to the host antibody. All the likelihoods are multiplied to get the final score.

Aside from the original sequences and the generated mutated sequences, we also generated random mutations by modifying the original sequences. With some fixed probability p, we selected whether a mutation would occur at our selected mutation sites. If the mutation occurs, then we defined the mutation as a random mutation with equal probabilities for all possible replacements of the residues.

12 Details of attention score extraction and aggregation

During inference, we extracted the attention scores for all the sequences. Additionally, we also extracted the weight each channel received while making the final prediction. We aggregated the attention scores in a weighted manner to calculate the final attention score. Finally, we extracted the top 7 sites from each timestep ranked by the attention score they received.

References

[1] Shu, Y. & McCauley, J. Gisaid: Global initiative on sharing all influenza data–from vision to reality. *Eurosurveillance* **22**, 30494 (2017).

[2] Cdc. Coronavirus disease 2019 (covid-19) (2020). URL https://web.archive.org/web/20220602002415/https://www.cdc.gov/coronavirus/2019-ncov/variants/variant-classifications.html.

[3] Angermueller, C., Lee, H. J., Reik, W. & Stegle, O. Deepcpg: accurate prediction of single-cell dna methylation states using deep learning. *Genome biology* **18**, 1–13 (2017).

[4] Sandaruwan, P. D. & Wannige, C. T. An improved deep learning model for hierarchical classification of protein families. *Plos one* **16**, e0258625 (2021).

[5] Kakati, T., Bhattacharyya, D. K., Kalita, J. K. & Norden-Krichmar, T. M. Degnext: classification of differentially expressed genes from rna-seq data using a convolutional neural network with transfer learning. *BMC bioinformatics* **23**, 17 (2022).

[6] Bileschi, M. L. *et al.* Using deep learning to annotate the protein universe. *Nature Biotechnology* **40**, 932–937 (2022).

[7] Elnaggar, A. *et al.* Prottrans: Towards cracking the language of lifes code through self-supervised deep learning and high performance computing. *IEEE Transactions on Pattern Analysis and Machine Intelligence* 1–1 (2021).

[8] Fukushima, K. Cognitron: A self-organizing multilayered neural network. *Biological cybernetics* **20**, 121–136 (1975).

[9] Bridle, J. Training stochastic model recognition algorithms as networks can lead to maximum mutual information estimation of parameters. *Advances in neural information processing systems* **2** (1989).

[10] Bao, W., Cui, Q., Chen, B. & Yang, B. Phage_unir_lgbm: phage virion proteins classification with unirep features and lightgbm model. *Computational and mathematical methods in medicine* **2022** (2022).

[11] Sharma, A. & Singh, B. Ae-lgbm: Sequence-based novel approach to detect interacting protein pairs via ensemble of autoencoder and lightgbm. *Computers in Biology and Medicine* **125**, 103964 (2020). URL https://www.sciencedirect.com/science/article/pii/S0010482520302973.

[12] Lv, Z., Wang, P., Zou, Q. & Jiang, Q. Identification of sub-Golgi protein localization by use of deep representation learning features. *Bioinformatics* **36**, 5600–5609 (2020). URL https://doi.org/10.1093/bioinformatics/btaa1074.

[13] Zhou, L. *et al.* Lpi-hyadbs: a hybrid framework for lncrna-protein interaction prediction integrating feature selection and classification. *BMC bioinformatics* **22**, 1–31 (2021).

[14] Liu, D., Huang, Y., Nie, W., Zhang, J. & Deng, L. Smalf: mirna-disease associations prediction based on stacked autoencoder and xgboost. *BMC bioinformatics* **22**, 1–18 (2021).

[15] Ben Or, G. & Veksler-Lublinsky, I. Comprehensive machine-learning-based analysis of microrna–target interactions reveals variable transferability of interaction rules across species. *BMC bioinformatics* **22**, 1–27 (2021).

[16] Yu, B. *et al.* SubMito-XGBoost: predicting protein submitochondrial localization by fusing multiple feature information and eXtreme gradient boosting. *Bioinformatics* **36**, 1074–1081 (2019). URL https://doi.org/10.1093/bioinformatics/btz734.

[17] Reddi, S. J., Kale, S. & Kumar, S. On the convergence of adam and beyond. *arXiv preprint arXiv:1904.09237* (2019).

[18] Thadani, N. N. *et al.* Learning from prepandemic data to forecast viral escape. *Nature* 1–8 (2023).

(a) Euclidean Distance

Table (8) Pairwise comparison of Euclidean distances and cosine dissimilarity between clusters

Actual Sequences \ Generated Sequences	Cluster 1	Cluster 2	Cluster 3	Cluster 4	Cluster 5	Cluster 6	Cluster 7
Cluster 1	**0.200**	-	-	0.854	1.423	0.324	1.402
Cluster 2	2.709	-	-	2.677	2.819	2.661	2.737
Cluster 3	0.202	-	-	0.857	1.424	0.329	1.403
Cluster 4	0.840	-	-	**0.411**	0.826	0.637	0.891
Cluster 5	1.192	-	-	0.571	**0.656**	0.987	0.761
Cluster 6	0.391	-	-	0.592	1.165	**0.250**	1.178
Cluster 7	1.275	-	-	0.721	0.689	1.100	**0.639**

(b) Cosine Dissimilarity

Actual Sequences \ Generated Sequences	Cluster 1	Cluster 2	Cluster 3	Cluster 4	Cluster 5	Cluster 6	Cluster 7
Cluster 1	**0.006**	-	-	0.124	0.327	0.016	0.324
Cluster 2	0.840	-	-	0.874	0.913	0.840	0.870
Cluster 3	0.007	-	-	0.125	0.327	0.017	0.324
Cluster 4	0.123	-	-	**0.033**	0.117	0.077	0.143
Cluster 5	0.241	-	-	0.060	**0.072**	0.176	0.100
Cluster 6	0.024	-	-	0.067	0.236	**0.011**	0.248
Cluster 7	0.268	-	-	0.091	0.079	0.210	**0.067**
